# GASTRIC AND JEJUNAL HISTOPATHOLOGICAL CHANGES IN PATIENTS UNDERGOING BARIATRIC SURGERY

**DOI:** 10.1590/0102-6720201600S10010

**Published:** 2016

**Authors:** Rosemary Simões Nomelini RODRIGUES, Élia Cláudia de Souza ALMEIDA, Silvia Maria Perrone CAMILO, Júverson Alves TERRA-JÚNIOR, Lucinda Calheiros GUIMARÃES, Ana Cristina da Rocha DUQUE, Renata Margarida ETCHEBEHERE

**Affiliations:** Universidade Federal do Triângulo Mineiro (Triângulo Mineiro Federal University - UFTM), Uberaba, MG, Brazil

**Keywords:** Obesity, Bariatric Surgery, Histology, Gastritis, Helicobacter pylori

## Abstract

**Background:**

Morbid obesity is a multifactorial disease that increasingly is being treated by surgery.

**Aim:**

To evaluate gastric histopathological changes in obese, and to compare with patients who underwent gastrojejunal bypass and the jejunal mucosa after the surgery.

**Methods:**

This is an observational study performed at a tertiary public hospital, evaluating endoscopic biopsies from 36 preoperative patients and 35 postoperative.

**Results:**

In the preoperative group, 80.6% had chronic gastritis, which was active in 38.9% (77.1% and 20.1%, respectively, in the postoperative). The postoperative group had a significant reduction in H. pylori infection (p=0.0001). A longer length of the gastric stump and a time since surgery of more than two years were associated with Helicobacter pylori infection. The jejunal mucosa was normal in 91.4% and showed slight nonspecific chronic inflammation in 8.6%.

**Conclusion:**

There was a reduction in the incidence of Helicobacter pylori infection in the postoperative group. A longer length of the gastric stump and longer time elapsed since surgery were associated with Helicobacter pylori infection. The jejunal mucosa was considered normal in an absolute majority of patients.

## RESUMO

**Racional:**

A obesidade mórbida é doença multifatorial cujo tratamento cirúrgico é cada vez mais indicado.

**Objetivo:**

Avaliar alterações histopatológicas gástricas em obesos e comparar com os submetidos à bypass gastrojejunal e a mucosa jejunal após a operação.

**Método:**

Estudo observacional realizado em hospital público terciário avaliando biópsias endoscópicas de 36 pacientes no pré-operatório e 35 no pós-operatório.

**Resultados:**

No pré-operatório 80,6% apresentaram gastrite crônica, 38,9% em atividade (77,1% e 20,1%, respectivamente, no pós-operatório). O grupo pós-operatório apresentou diminuição significativa na infecção por Helicobacter pylory (p=0,0001). Maior comprimento do coto gástrico e tempo de operação superior a dois anos associaram-se a infecção por Helicobacter pylori. A mucosa jejunal foi normal em 91,4% e apresentava leve inflamação crônica inespecífica em 8,6%.

**Conclusão:**

Houve diminuição da infecção por Helicobacter pylori após a operação. Maior comprimento do coto gástrico e do tempo de operação associaram-se à infecção por Helicobacter pylori. A mucosa jejunal foi considerada normal na maioria absoluta dos pacientes do grupo pós-operatório.

## INTRODUCTION

Obesity is a chronic disease characterized by excessive accumulation of adipose tissue in the organism. This disease has increased in prevalence in recent decades, particularly in developing countries, and the levels have nearly doubled between 1980 and 2014^6, 10, 28^. Obesity has a multifactorial etiology that depends on interactions among genetic, metabolic, social, behavioral, and cultural factors^19^. Obesity requires a multi-level approach for treatment, with dietary guidance, regular physical activity, and drugs being the main pillars of this treatment. However, conventional treatment for morbid obesity produces unsatisfactory results, with around 95% of patients regain to their initial weight within two years of treatment. Accordingly, bariatric surgery is increasingly being indicated for this condition^26^. Objectives of bariatric surgery, aside from weight loss, are a reduction in the presence of comorbidities and an improvement in the patient's quality of life^17^_._

Despite the increased performance of bariatric surgery in recent years, few studies have investigated the histologic changes in the gastric mucosa of obese patients before surgery and in the mucosa of the Roux-en-Y anastomosis after surgery^1^ ^,^ ^22^ ^,^ ^24^. 

Therefore, the objectives of this study were to evaluate histopathologic changes to the gastric mucosa in obese patients who were about to undergo bariatric surgery, and to compare these changes to the gastric and jejunal changes found in patients who had undergone surgery at least one year earlier.

## METHODS

This observational study was approved by the institution's ethics committee and performed at a tertiary public hospital from April 2014 to July 2015. Were evaluated 36 patients in the preoperative period before bariatric surgery (preoperative group) and 35 patients who had undergone surgery by the gastroduodenal bypass technique with Roux-en-Y reconstruction at least one year earlier (postoperative group). Patients who agreed to participate signed an informed consent form. Inclusion criteria were morbid obesity (BMI >40 kg/m^2^) and an indication for bariatric surgery or previous duodenal bypass with Roux-en-Y reconstruction performed at least one year earlier. Exclusion criteria were malformations or previous surgery in the upper gastrointestinal tract.

Were collected clinical and demographic data, such as gender, presence of comorbidities (e.g., diabetes mellitus, systemic hypertension, or depression), and BMI, during an interview before performing upper gastrointestinal endoscopy (UGIE). All patients underwent UGIE, which was performed using an Olympus videoendoscope device (GIF-Q150^(r)^ and GIF-2T160^(r))^ with an Exera-CLV-160 processor. During the exam, were collected biopsies of the gastric body from preoperative patients and biopsies of the gastric stump and jejunal mucosa from postoperative patients. Was chosen to evaluate the gastric body (oxyntic mucosa) in the preoperative group to enable comparisons with the gastric stump, which is usually of this type^25^. 

Biopsies were fixed in 4% buffered formalin, processed, and encased in paraffin. They were stained with hematoxylin and eosin for general evaluation and with the Warthin-Starry stain for *Helicobacter pylori* (HP) research. Were evaluated biopsies for the presence or absence of the following criteria: erosion/ulceration, scarring, lymphatic follicles, mononuclear and polymorphonuclear inflammatory infiltrates (inflammatory activity), glandular body hypotrophy, intestinal metaplasia, reactive gastropathy, and bacteria that are morphologically compatible with HP. When applicable, the intensity of the features was quantified as absent, slight, moderate, or intense, as proposed by the 1996 Sidney Consensus^12^ ^,^ ^13^. A single medical pathologist analyzed all biopsies.

There was no statistical calculation to define the sample size, which was defined by accessibility because of the difficulties in making up the postoperative group. Results were entered into a database by using Microsoft Access 2000^(r)^ and statistically analyzed by using the Biostat^(r)^ program (version 5.0). Were applied the Fisher exact and Mann-Whitney tests, which were considered significant when the probability of rejecting the hypothesis was lower than 5% (p< 0.05).

## RESULTS

Table 1 summarizes the demographic characteristics of patients in the two groups. In the preoperative group, only 40.0% of patients had normal findings on UGIE. The remaining 60% had erosive or non-erosive gastritis (54.3%), esophagitis (14.3%), duodenitis (11.4%), or polyp (8.6%).

**TABLE 1:** Demographic and clinical information of patients in the pre- and postoperative groups for bariatric surgery

	Group	
Characteristics	Preoperative (n=36)	Postoperative (n=35)
Age	40 years old (md=23 - 58)	45 years old (md= 29 - 64)
Sex	30 women (83.3%) 6 men (16.7%)	31 women (88.6%) 4 men (11.4%)
BMI (kg/m^2^)	45.3±5.2 (37.0-62.7)	29.9±5.4 (24.9-46.0) *
SH	66.7%	17.1% **
DM	41.7%	11.4% ***
Osteoarticular disorder	16.7%	2.9%
Depression	25.0%	20.0%

BMI=body mass index; SH=systemic hypertension; DM=diabetes mellitus. * p< 0.0001; **p=0.0000; ***p=0.004

The time since surgery in the postoperative group ranged from 1 to 15 years (median: 7 years), with 17.1% of patients having a time since surgery between 1 and 2 years, 14.3% between 2 and 5 years, and 60% of 5 or more years. The length of the remaining gastric stump ranged from 3 to 10 cm. The length was shorter than 4 cm in 2.9% of patients; between 4 and 6 cm in 65.7% of patients; and longer than 6 cm in 31.4% of patients. On UGIE, 91.4% of postoperative patients had description of normal gastric stump and jejunum mucosa.

Table 2 shows the histopathological findings in the oxyntic gastric mucosa for the preoperative and postoperative groups. In the preoperative group, 80.6% of patients had chronic inflammation of the oxyntic gastric mucosa, which was classified as slight (44.4%), moderate (30.6%), or intense (5.6%). Inflammatory activity was present in 38.9% of preoperative patients, classified as slight in 25%, moderate in 5.6%, and intense in 8.3% of patients. HP infection was present in 63.9%. In the postoperative group, 77.1% of patients had chronic gastritis, which was classified as slight (57.1%), moderate (17.1%), or intense (2.9%). Inflammatory activity was present in 20.1% of postoperative patients, and was classified as slight in 8.6%, moderate in 8.6%, and intense in 2.9% of patients. HP infection was present in 28,6%.

**TABLE 2:** Histopathological findings in the oxyntic gastric mucosa for patients in the pre- and postoperative groups

	Preoperative	Postoperative
	(n=36)	(n=35)
Erosion/ ulceration	0	0
Scarring	0	0
Lymphoid follicle	8.3%	22.9%
Chronic inflammation	80.6%	77.1%
Inflammatory activity	38.9%	20.0%
Hypotrophy	0	5.7%
Intestinal metaplasia	0	5.7%
Reactive gastropathy	0	0
Helicobacter pylori	63.9%	28.6% *

*p=0.002

**FIGURE 1:**
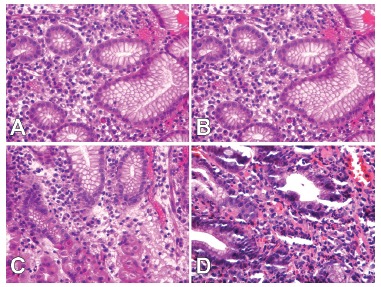
Histological slices of oxyntic mucosa in the pre- (A) and postoperative groups (B), with chronic gastritis characterized by a large quantity of plasma cells on the corion; C and D show the inflammatory activity, characterized by the permeation of the epithelium by neutrophils (hematoxylin and eosin, 400×).

Representative histologic sections for the two groups are provided in Figure 1.

**FIGURE 2:**
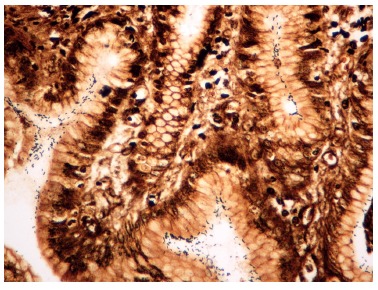
Histological slices of oxyntic gastric mucosa in the postoperative group, showing numerous spiraled bacteria (in black) on the surface of the epithelium and in the interior of the crypt compatible with HP infection (Wartin-Starry, 400×).

One HP-positive case had a residual stump length smaller than 4 cm (10%), four cases had a stump length between 4 and 6 cm (40%), and five cases had a stump length exceeding 6 cm (50%). Statistical analysis showed a significant relationship (p= 0.0001), suggesting that stumps with a length greater than 6 cm were more often associated with HP infection (Figure 2).

Were compared the time since surgery with the presence of HP infection. None of the patients who had undergone surgery in the previous two years were infected with HP. In contrast, HP infections were found in five patients who underwent surgery between two and five years earlier, and in five patients who underwent surgery more than five years earlier. Statistical analysis showed a significant relationship (p=0.0014), suggesting that a time since surgery of two or more years was more often associated with HP infection. 

When comparing the BMI ≤30 kg/m^2^ and ≥35 kg/m^2^ in the postoperative group with HP infection, was found no statistically significant difference (p=0.5835 and 0.6879, respectively).

Finally, when was analyzed the jejunal Roux-en-Y mucosa, 91.4% of postoperative patients had mucosa that could be considered histologically normal. The remaining 8.6% of patients had slight, nonspecific chronic inflammation.

## DISCUSSION

Obesity is a multifactorial chronic disease that is increasingly being treated with surgery^6^ ^,^ ^10^ ^,^ ^19^ ^,^ ^28^. Consistent with the literature we found elevated levels of comorbidities in obese patients in the preoperative period^10^ ^,^ ^17^ ^,^ ^23^. After surgery, as expected, there was a significant reduction in BMI and in the incidence of comorbidities, such as diabetes mellitus and systemic hypertension. We frequently observed chronic gastritis in both groups. Infection with HP, the main cause of gastritis^4^ was significantly more common in preoperative than in postoperative patients. This bacterium is very prevalent worldwide. In a study performed in Brazil, Ddine et al.^11^ observed HP infection in around 18.5% of obese patients, a lower frequency than was observed in this study. Yang et al.^29^ analyzed patients who had undergone vertical gastroplasty or Roux-en-Y gastric bypass and observed HP infection in both symptomatic (39%) and asymptomatic (39.7%) patients after surgery.

Obesity, gastritis, and HP infection are not necessarily associated, although studies suggest that HP infection can affect food consumption through ghrelin, a peptide secreted in the stomach. There is a physiological rise in ghrelin levels during fasting, which increases appetite. Eating causes a reduction in ghrelin secretion and, consequently, of appetite. HP-induced gastritis can lead to reductions in ghrelin levels and body mass. Therefore, eradication of the bacteria normalizes ghrelin levels, increasing bodyweight. However, this idea remains controversial. Some studies have indicated a weight gain with infection, while others have not observed a change in ghrelin levels with HP infection^5^ ^,^ ^14^. Wang et al.^27^ observed that patients infected with HP and who have gastritis exhibit significantly less weight loss when tracked for 24 to 48 months after surgery. Our findings indicated no association between HP infection and higher BMI in postoperative group.

A reduction in the occurrence of HP infection in patients who undergo an operation might be explained by the treatment protocol, which seeks to eradicate the bacteria before surgery and, thus, avoid surgical complications^3^ ^,^ ^18^. In gastrojejunal bypass, the Roux-en-Y is made into a pouch (gastric stump), dominated by the greater curvature and, sometimes, limited to the cardia, which reduces the population of parietal cells. However, Siilin et al.^25^ considered it to be practically impossible to make a pouch that does not contain parietal cells. These technical aspects lead to a reduction in the occurrence of marginal ulcer and HP infection. Csendes et al.^7^ found that HP infection was present in 46.8% of patients before gastroplasty compared to 31% after surgery. Of the patients with HP infection after surgery, 50% were already carriers of the bacteria before surgery. The authors suggest that despite there being few parietal cells in the gastric pouch, HP was able to colonize it anew. An interesting finding of the present study is that patients who had undergone surgery more than two years previously had a significant increase in HP infection. This result suggests that the passage of time increases the chances that HP will recolonize the mucosa. 

Studies after partial gastrectomy and Roux-en-Y anastomosis for benign disease demonstrated there are a total of 41% of patients presented HP reinfection at the gastric stump, which increased parallel to the length of follow-up^8^. We observe much lower percentage in our study. A possible explanation for this difference is small gastric stump after gastric bypass left in bariatric surgery, as compared to surgery performed for benign disease (gastric pouch with the remaining eight to ten times greater)^8^. Agreeing with this impression, we find significant difference in the length of residual gastric stump with HP infection, suggesting that stumps larger than 6 cm would be more associated with this infection. However, further studies are needed. On the other hand, it is not fully established the colonization of the gastric stump by HP after bypass is associated with complications^8^ ^,^ ^18^. Evaluation of the stump size by UGIE is a subjective evaluation that is influenced by the surgical technique. Previous authors have suggested ideal stump sizes ranging from 1.8 to 8.0 cm^2,^ ^16^. Moreover, our study included very few patients with a gastric stump smaller than 3 cm. Other authors, analyzing the gastric pouch and the presence of HP, have found no association between the presence of HP and the gastric pouch or gastrojejunal anastomosis size. These authors concluded that the behavior of HP is inconsistent and difficult to interpret^9^.

Levels of gastritis in the gastric stump vary in the literature. We observed chronic gastritis, usually slight, in the majority of cases. Marano^20^ found normal endoscopic results in 30% of postoperative patients, although all patients who underwent UGIE were symptomatic. On the other hand, Marcuard et al.^21^ reported acute or chronic gastritis in all patients. Flickinger et al.^15^ performed UGIE on patients 13 to 20 months after surgery, and the pouch was described as endoscopically normal in 85% of patients. However, histology showed normality in only 45%, acute gastritis in 23%, chronic gastritis in 30%, and intestinal metaplasia in 13% of patients. Our percentage of patients with chronic gastritis was higher and that of intestinal metaplasia lower than those reported in this previous study. In addition, we did not find any cases of acute gastritis. When analyzing the gastric pouch two years after surgery, other authors showed endoscopic normality in 99% of patients. Histology was normal in 56%, gastritis was present in 28.1%, and intestinal metaplasia was present in 4.0% of patients. Csendes et al.^7^ also studied the mucosa of the intestinal loop, which was normal in all patients, both macroscopically and histologically. Our findings were similar to those described by these authors.

## CONCLUSION

Was observed a significant reduction in the percentage of patients with HP infection after bariatric surgery. A residual gastric stump length exceeding 6 cm and a time since surgery exceeding two years were associated with a higher rate of HP infection. These findings indicated no association between HP infection and higher BMI in postoperative group. The jejunal mucosa was considered normal in an absolute majority of patients.
